# Chemical/Instrumental Approaches to the Evaluation of Wine Chemistry

**DOI:** 10.3390/molecules25061363

**Published:** 2020-03-17

**Authors:** Rosa Perestrelo, José S. Câmara

**Affiliations:** CQM—Centro de Química da Madeira, University of Madeira, 9020-105 Funchal, Portugal

Wine is a widely consumed beverage thanks to its unique and pleasant sensory properties. Wine is composed of more than one thousand chemical compounds (e.g., alcohols, esters, acids, terpenoids, phenolic compounds, flavonoids, anthocyanins, minerals, vitamins, among others) resulting from several chemical and biochemical processes [1,2]. Nowadays, microextraction techniques tandem with high-resolution analytical instruments have been applied by wine researchers to expand the knowledge of wine´s chemical composition with the purpose to improve wine quality, support winemaker decisions related to the winemaking process, and guarantee the authenticity and genuineness of wine [3,4,5,6].

As a result, we proposed “Chemical/Instrumental Approaches to the Evaluation of Wine Chemistry” as an interesting topic for a Special Issue in the Molecules journal. This Special Issue aims to update the top-of-the-art extraction procedures (e.g., solid-phase microextraction (SPME)) and analytical tools (e.g., nuclear magnetic resonance (NMR), inductively coupled plasma mass spectrometry (ICP-MS), ultra-performance liquid chromatography tandem mass spectrometry (UPLC-MS/MS)), emphasizing their use as suitable platforms for the establishment of the chemical composition of wine (volatomic profile, antioxidants, phenolic pattern, elemental composition, among others). In addition, information related to wine sensorial properties, contaminants, authenticity, and chemometric tools used for data treatment will be described in this issue. Thus, this Special Issue includes eight publications using different analytical approaches for the evaluation of wine chemistry [7,8,9,10,11,12,13,14]. Regarding gas chromatography, Sancho-Galán et al. [11] used gas chromatograph equipped with a flame ionization detector (GC-FID) to study the use of bee pollen as a flor velum activator in biological aging wines. Moreover, headspace solid-phase microextraction combined with gas chromatography-mass spectrometry (HS–SPME/GC–qMS) was used by Dang et al. [14] for determining the retention of volatile phenols (putative markers for Brettanomyces and smoke taint off-odors) by cyclodextrin in model wine, as well as by Perestrelo el al. [7] to investigate the volatile organic compounds (VOCs) that may potentially be responsible for specific descriptors of Madeira wine, providing details about Madeira wine aroma notes at the molecular level. 

Related to liquid chromatography, Tarapatskyy et al. [13] used ultra-performance reverse-phase liquid chromatography tandem mass spectrometry (UPLC-MS/MS) to assess the bioactive compounds in white and red wines enriched with a *Primula veris* L. In addition, a novel and accurate method based on ultrahigh performance liquid chromatography (UHPLC) with a photo-diode array detector (PDA) and charged aerosol detector (CAD) was developed for simultaneously determining nine sweeteners (most authorized for use in China) in white spirits by Ma et al. [8].

Deng et al. [10] used inductively coupled plasma mass spectrometry (ICP-MS) to determine the concentration of trace elements in wines and health risk assessment via wine consumption was investigated in 315 wines. In this context, Tamasi et al. [9] used ionic exchange resins and hydrogels for capturing metal ions (Na, K, Mg, Ca, Mn, Fe, Cu and Zn)) in sweet dessert wines. Moreover, Li et al. [12] used a nanoparticle tracking analysis (NTA) and UV-visible spectroscopy and dynamic light scattering (DLS) to characterize the interactions between grape seed tannin and either a mannoprotein or an arabinogalactan in model wine solutions of different ethanol concentrations. 

This Special Issue is accessible through the following link:

https://www.mdpi.com/journal/molecules/special_issues/instrumental_wine_chemistry

As Guest Editors for this Special Issue, we would like to thank all the authors and co-authors for their contributions, all reviewers for their effort in revising the manuscripts, as well as the editorial office of Molecules journal for their generous help in organizing this Special Issue.
